# Neonatal vitamin D status and asthma risk after age 5 years: A Danish population‐based cohort study

**DOI:** 10.1111/pai.70299

**Published:** 2026-02-02

**Authors:** Xiaoqin Liu, Zhihong Zhu, Bo Chawes, Klaus Bønnelykke, Henriette Thisted Horsdal, Esben Agerbo, Sanne Grundvad Boelt, Nis Borbye‐Lorenzen, Lars Melgaard, Carsten Bøcker Pedersen, John J. McGrath

**Affiliations:** ^1^ NCRR‐The National Centre for Register‐Based Research, Department of Public Health Aarhus University Aarhus Denmark; ^2^ COPSAC, Copenhagen Prospective Studies on Asthma in Childhood, Herlev and Gentofte Hospital University of Copenhagen Copenhagen Denmark; ^3^ Department of Clinical Medicine, Faculty of Health and Medical Science University of Copenhagen Copenhagen Denmark; ^4^ Integrated Register‐Based Research Aarhus University Aarhus Denmark; ^5^ Big Data Center for Environment and Health Aarhus University Aarhus Denmark; ^6^ Danish Center for Neonatal Screening, Department of Congenital Disorders, Statens Serum Institut Copenhagen Denmark; ^7^ Queensland Brain Institute University of Queensland Brisbane Queensland Australia

**Keywords:** asthma, cohort study, neonatal, polygenic score, population‐based, registers, vitamin D

## Abstract

**Background:**

Vitamin D may play a role in early lung development, yet epidemiologic evidence on its association with later asthma risk is mixed. We aimed to investigate the associations of neonatal 25‐hydroxyvitamin D (25(OH)D) and vitamin D‐binding protein (DBP) and their corresponding genetic predictors with asthma risk.

**Methods:**

We conducted a population‐based cohort study of a random sample of individuals born in Denmark during 1991–2005 from the iPSYCH2012 study. Neonatal concentrations of 25(OH)D and DBP were measured via dried blood spots, and asthma cases were identified through diagnoses or asthma prescriptions after age 5 years. Cox regression was used to estimate hazard ratios (HRs) for asthma in relation to 25(OH)D, DBP, and polygenic scores (PGSs) for these traits and asthma to assess genetic liability to vitamin D status and asthma.

**Results:**

Of 14,005 individuals, 2308 (16.5%) developed asthma over a maximum follow‐up of 25 years. We found no association between neonatal 25(OH)D (HR = 1.04, 95% CI: 0.99–1.09 per SD increase) or DBP (HR = 1.01, 95% CI: 0.97–1.05) and asthma risk. Analyses using tertiles to assess potential non‐linear associations yielded similar null results. A higher asthma PGS was associated with increased asthma risk (HR = 1.42, 95% CI: 1.36–1.47 per SD increase), whereas PGSs for 25(OH)D (HR = 1.00, 95% CI: 0.96–1.05) and DBP (HR = 0.99, 95% CI: 0.95–1.04) were not.

**Conclusions:**

Our study suggests that neonatal vitamin D status is not associated with asthma risk. Similarly, genetic liability related to vitamin D status, as reflected in PGSs for 25(OH)D and DBP, is not associated with an increased risk of asthma.

**Key message**

The study provides robust population‐based evidence that neonatal vitamin D status, measured through 25(OH)D, DBP, and their polygenic predictors, is not associated with asthma risk. By integrating biomarker and genetic data from neonatal dried blood spots with long‐term follow‐up, our findings clarify the inconsistent literature and suggest that early‐life vitamin D levels are unlikely to play a major role in asthma development.

## 1 INTRODUCTION

Vitamin D is a prohormone that is acquired mainly via the action of sunlight on the skin, but also through dietary intake and supplements. However, lifestyle changes, such as reduced sun exposure and prolonged indoor activities, can lead to vitamin D deficiency or insufficiency. This condition affects approximately 45% of individuals globally,^1^ and can occur at any stage of life, with pregnant women and newborns being especially at high risk.^2^ The early stages of life represent a crucial development phase for the immune and respiratory systems.^3^ Given its influence on fetal lung cell maturation and subsequent lung function,^4^ deficiency/insufficiency of vitamin D at this critical period may lead to an increased risk of asthma/wheeze.^5^, ^6^

Epidemiological studies on asthma risk and cord blood or maternal 25(OH)D levels have shown inconsistent findings. Three studies suggested an inverse association between higher 25(OH)D levels and asthma risk^7^, ^8^, ^9^; two studies reported an increased asthma risk with higher maternal 25(OH)D levels,^10^, ^11^ whereas most did not find an association.^4^, ^12^, ^13^, ^14^, ^15^, ^16^, ^17^, ^18^, ^19^, ^20^, ^21^ Even meta‐analyses have shown mixed results: A meta‐analysis (encompassing 35,046 participants and 2785 cases) indicated a U‐shaped relationship between maternal 25(OH)D levels and childhood asthma,^22^ whereas another meta‐analysis showed that elevated cord blood 25(OH)D levels were associated with a reduced risk of early‐life wheeze (odds ratio for the highest vs. lowest 25(OH)D group = 0.43, 95% CI: 0.29–0.62).^23^ A recent Cochrane review found that vitamin D supplementation during pregnancy may reduce childhood wheeze but not asthma, while evidence for supplementation in early childhood remains inconclusive.^24^ Existing studies have been limited to short observation periods, primarily focusing on preschool children, with a maximum follow‐up of 14 years. Most studies have been based on small samples and may lack the power to detect a meaningful association.

The active form of 25(OH)D is bound to vitamin D‐binding protein (DBP). Higher DBP levels extend the half‐life of 25(OH)D and may affect the risk of vitamin D deficiency.^25^ Although genetic studies suggest an association between DBP variants and asthma susceptibility,^26^, ^27^ only one study has investigated neonatal DBP concentrations.^9^ This study reported a stronger protective effect of 25(OH)D at lower DBP levels; however, it used a simplistic approach to estimate free 25(OH)D, relying solely on the ratio of total 25(OH)D to DBP.^9^

Given these considerations, we conducted a large, population‐based cohort study in Denmark to examine the associations between neonatal 25(OH)D and DBP levels and the subsequent asthma risk. In contrast to prior studies, which were largely limited by small sample sizes, short follow‐up, or indirect exposure assessment, our study leveraged two neonatal biomarker measurements and up to 25 years of follow‐up. We hypothesized that lower 25(OH)D and DBP levels contributed to an elevated asthma risk. Our secondary analyses focused on genetic predictors of asthma, 25(OH)D, and DBP with respect to asthma risk.

## 2 METHODS

### 2.1 Setting

We conducted a population‐based cohort study linking the Integrative Psychiatric Research (iPSYCH) 2012 study sample^28^ with the Danish national registers. We reported the study in accordance with the Strengthening the Reporting of Observational Studies in Epidemiology (STROBE) guidelines. A detailed description of the iPSYCH 2012 study has been previously described.^29^ Briefly, the iPSYCH 2012 study was selected from the Danish Civil Registration System^30^ of all singleton births born between May 1, 1981, and December 31, 2005, who were alive and residing in Denmark at the age of 1 year and had a known mother. All individuals with a major mental illness by December 31, 2012, were identified as cases in the iPSYCH 2012 sample (*N* = 57,377). In addition, a random sample of 30,000 individuals was selected from the Danish population born at the same time (the subcohort) to facilitate future non‐psychiatric research questions, with a sampling probability of 2.04%. This subcohort is representative with respect to key demographic characteristics of the underlying population.^28^

The Danish Newborn Screening Biobank stored dried blood spots taken at birth from nearly all infants born in Denmark since May 1, 1981.^31^ The Danish Prescription Registry was established in 1995 and contains information on all prescribed drugs dispensed at pharmacies in the Danish community.^32^ Medications prescribed for individuals under 16 years were registered as dispensed for their mother before January 1, 1996, and subsequently registered under the child's own personal identification number. The Danish National Patient Register contains information on inpatients since 1977, and outpatients and emergency room visits since 1995.^33^

### 2.2 Study population

**FIGURE 1:**
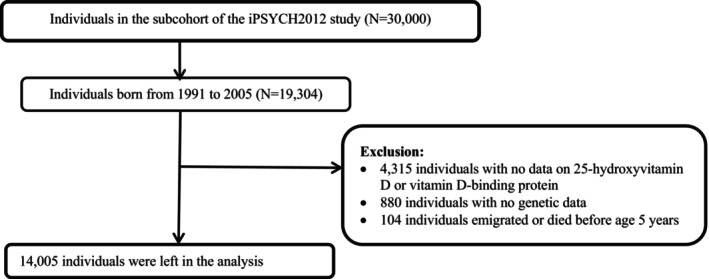
Flowchart of the study population.

We included 19,304 individuals born between January 1, 1991, and December 31, 2005, in the population‐based iPSYCH2012 subcohort (Figure 1 for the flowchart). We restricted our analyses to this subcohort to ensure population representativeness and to avoid potential selection related to the ascertainment of mental disorders. We included individuals born after January 1, 1991, to ensure valid data on filled prescriptions (comprehensively recorded since 1996) and hospital contacts were available for them at 5 years. We excluded 4315 individuals with no data on neonatal levels of 25(OH)D and DBP, 880 individuals with no genetic data, and 104 individuals who emigrated or died before age 5 years to ensure we could retrieve information on asthma diagnosis after age 5 years, leaving 14,005 individuals in the final analyses. Overall, individuals included and excluded from the study were largely comparable with respect to maternal age, primiparity, cohabitation status, highest educational attainment, and maternal asthma, as well as gender of the individuals. However, compared with included individuals, a higher proportion of those born in (a) earlier calendar years and (b) spring and autumn, were in the excluded group (Table S1).

### 2.3 Ascertainment of asthma—Outcome of interest

We considered an individual to have asthma if they fulfilled at least *one* of the three well‐established algorithms (A–C)^34^, ^35^ after age 5 years:

(A) At least one hospital contact for asthma (International Classification of Diseases, 10th Revision (ICD‐10) codes J45, J45.0, J45.1, J45.8, J45.9, and J46.9) in the Danish National Patient Register.^33^

(B) At least two prescriptions for asthma medication within 12 months in the Danish National Prescription Registry,^32^ based on the following ATC codes: Inhaled short‐acting β2‐agonists (R03AC02–04), inhaled long‐lasting β2‐agonists (R03AC12, R03AC13, and R03CC12), inhaled glucocorticoids/corticosteroids (R03BA01, R03BA02, and R03BA05), fixed‐dose combination of inhaled long‐lasting β2‐agonists and glucocorticoids (R03AK06 and R03AK07), leukotriene receptor antagonists (R03DC03), rarely used asthma medications (R03BC01, R03AK03, R03AK04, R03BB01, R03BB04, and R03DA04), and new asthma medications since 2007 (inhaled glucocorticoids/corticosteroids, and combination of inhaled long‐lasting β2‐agonists and glucocorticoids: R03BA07, R03BA08, R03AK08, R03AK10, R03AK11, and R03AK14).

(C) At least two prescriptions of the following two asthma medications with no requirement of two prescriptions within 12 months: Inhaled corticosteroids (R03AK06–14 and R03BA) and leukotriene antagonists (R03DC03).

The diagnosis date of asthma was defined as the date of the second prescription filled for asthma medication, defined in algorithms B and C, or the first hospital contact for asthma, whichever occurred first. We restricted our analyses to those with asthma after age 5 years, as an accurate diagnosis of asthma in younger children can be challenging.

### 2.4 Measures of 25(OH)D and DBP concentrations

The 25(OH)D and DBP concentrations, as well as genotypes, were analyzed in neonatal dried blood spots collected during routine screening at birth in Denmark since 1981.^31^ Briefly, for the measurement of 25(OH)D, we adapted previously published methods based on tandem mass spectrometry.^36^, ^37^ The measurement was validated through both external and internal methods.^36^ We evaluated Standard Reference Material 972 serum from the National Institute of Standards and Technology (Gaithersburg, MD, USA) and found excellent accuracy, with results ranging from 92% to 105%. Our method detected concentrations of 25(OH)D as low as approximately 5 nmol/L in the protein extracts. All analyses were based on the total 25(OH)D, which includes the sum of 25(OH)D2 and 25(OH)D3. During the study period, our laboratory participated in the Vitamin D External Assessment Scheme, which confirmed the accuracy of our assay platform.

For the measurement of DBP levels, proteins extracted from dried blood spots were analyzed using a multiplex immunoassay with U‐plex plates from Meso‐Scale Diagnostics (MSD), located in Maryland, USA. We used antibodies specific to DBP (HYB249–05 and HYB249–01) sourced from Statens Serum Institut Antibodies in Copenhagen, Denmark. The detection limits for DBP were 2.07 μg/L for the lower limit and 79.8 mg/L for the upper limit. The intra‐assay variation was 7.6%, while the inter‐assay variation was 22.4%.^38^

For both 25(OH)D and DBP levels, we accounted for plate/batch effects by regressing them out of the estimates using a mixed linear model. Both neonatal 25(OH)D and DBP concentrations were non‐normally distributed and skewed, so we then transformed the data using rank‐based inverse normal transformation (RINT) to normalize the distributions and improve model fit. This approach is consistent with our previously published work using the same data.^39^

### 2.5 Covariates

The following covariates were identified a priori based on causal diagrams: maternal age at delivery, European ancestry, parity, maternal hospital contact for asthma before delivery (ICD‐8 code 493, ICD‐10 codes J45, J45.0, J45.1, J45.8, J45.9, J46.9), cohabiting status at delivery, highest education attained, season of birth, gender of the individuals, and calendar year of birth. European ancestry was determined based on the offspring's genotype, as described by Privé et al.^40^ In brief, the first 20 principal components (PCs) will be estimated from common single‐nucleotide polymorphisms (minor allele frequency ≥0.01), and European ancestry will be inferred using the PC‐based distance from cluster center (log_dist <4.8). We categorized the season of birth into winter (December to February), spring (March to May), summer (June to August), and fall (September to November).

### 2.6 Statistical analysis

Each individual was followed from age 5 years until the earliest occurrence of asthma, emigration, death, or December 31, 2021. The start and end of follow‐up are illustrated in the Lexis plot provided in Figure S1. We analyzed concentrations of 25(OH)D and DBP both as continuous variables (per standard deviation (SD) increase) and as tertiles to account for potential non‐linear associations, in separate models. Kaplan–Meier curves were used to illustrate the cumulative incidence of asthma by the tertiles of 25(OH)D and DBP. We performed Cox proportional hazards regressions to estimate the hazard ratios (HRs) and 95% confidence intervals (95% CIs) for asthma. The underlying time scale was the age of the individuals. Proportionality was evaluated by visually inspecting “log–log” plots. Only 2.0% of individuals had missing data on the highest level of maternal education attained, and these were included as a separate category. Statistical analyses were performed using STATA 18.0 (StataCorp, College Station, TX, USA).

We conducted two secondary analyses: First, we examined the association of PGSs for asthma, 25(OH)D, and DBP and asthma risk. Second, we assessed whether associations of 25(OH)D and DBP with asthma were modified by genetic susceptibility to asthma, using additive interaction models and estimating the Relative Excess Risk due to Interaction (RERI). DNA genotyping was conducted at the Broad Institute (Boston, MA, USA) using the Infinium PsychChip (version 1.0 array; Illumina, San Diego, CA, USA). We derived the PGS by weighting the effect size of multiple risk alleles obtained from genome‐wide association studies (GWAS),^41^ using the LDpred2‐auto approach.^42^ We used the same approach to estimate the PGSs of 25(OH)D and DBP.^39^ We standardized the PGSs using the mean and SD of each PGS: (observed value – mean)/SD. For these two secondary analyses, we further adjusted for the first 10 principal components.^40^

We conducted two sensitivity analyses to test the robustness of our results. First, skin type may influence vitamin D synthesis, and variations in DBP are associated with non‐European ancestry. We repeated our analyses specifically for individuals of European and non‐European ancestry. Second, we examined the relationships between 25(OH)D and DBP and childhood‐onset asthma (diagnosed by age 20 years) and adulthood‐onset asthma separately.

As a post‐hoc analysis, we investigated the relationship between neonatal 25(OH)D levels and DBP levels and risk of transient wheezing before age 5 years, among 9727 individuals born between 1996 and 2005, for whom prescription data were available from birth. Transient wheezing was defined as an individual meeting at least one of the criteria A–C before age 5 years but not meeting any of the criteria A–C after age 5 years.

#### 2.6.1 Statistical power

With 14,005 individuals, approximately 4670 were included in each tertile group. Based on a 5% significance level and an observed asthma risk of 19%, our study had ≥80% power to detect a hazard ratio of 0.87 or lower, corresponding to a minimum detectable risk reduction of 13% in higher tertiles compared to the lowest.

#### 2.6.2 Ethical considerations

The analyses of neonatal samples from the Danish Neonatal Screening Biobank presented in this study were approved by the steering committee of the Danish Neonatal Screening Biobank as well as the regional Scientific Ethical Committee of Mid Jutland. This study has been approved by the Danish Data Protection Agency and the Danish Health Data Authority. Under Danish law, informed consent is not required for register‐based studies that utilize anonymized data.

## 3 RESULTS

Of the 14,005 individuals, 86.9% were of European ancestry. The overall raw mean (SD) for 25(OH)D was 23.60 (14.01) nmol/L, and the overall raw mean for DBP was 2.21 (1.45) mg/L. The distribution of neonatal 25(OH)D and DBP after rank‐based inverse normal transformation can be seen in Figures S2 and S3. Characteristics of the individuals are presented in Table 1 and Table S2, stratified by neonatal 25(OH)D tertiles, and in Table S3, stratified by DBP tertiles. Over a maximum follow‐up of 25 years (interquartile range: 12.7–20.9 years), 2308 individuals (16.5%) developed asthma, corresponding to a cumulative incidence of 19.0% (95% CI: 18.2%–19.9%).

**TABLE 1:** Demographics of the study population (*N* = 14,005).

Maternal characteristics	*N* (%)
Maternal age at delivery	
<25	2392 (17.1)
25–29	5220 (37.3)
30–34	4523 (32.3)
≥35	1870 (13.4)
Primiparous	6172 (44.1)
Maternal cohabitant status	
Married or cohabiting	12,960 (92.5)
Single, divorced, or widowed	1045 (7.5)
Maternal highest education attained	
Mandatory school	3368 (24.0)
High school or vocational school	6338 (45.3)
College or university	4019 (28.7)
Unknown	280 (2.0)
Maternal asthma hospital diagnosis before delivery	225 (1.6)
European ancestry	
Yes	12,167 (86.9)
No	1838 (13.1)
Season of birth	
Spring	3498 (25.0)
Summer	3808 (27.2)
Autumn	3281 (23.4)
Winter	3418 (24.4)
Gender of the individuals	
Males	7154 (51.1)
Females	6851 (48.9)
Calendar year at birth	
1991–1995	4278 (30.5)
1996–2000	4840 (34.6)
2001–2005	4887 (34.9)

### 3.1 Neonatal 25(OH)D and DBP levels with asthma risk

**FIGURE 2:**
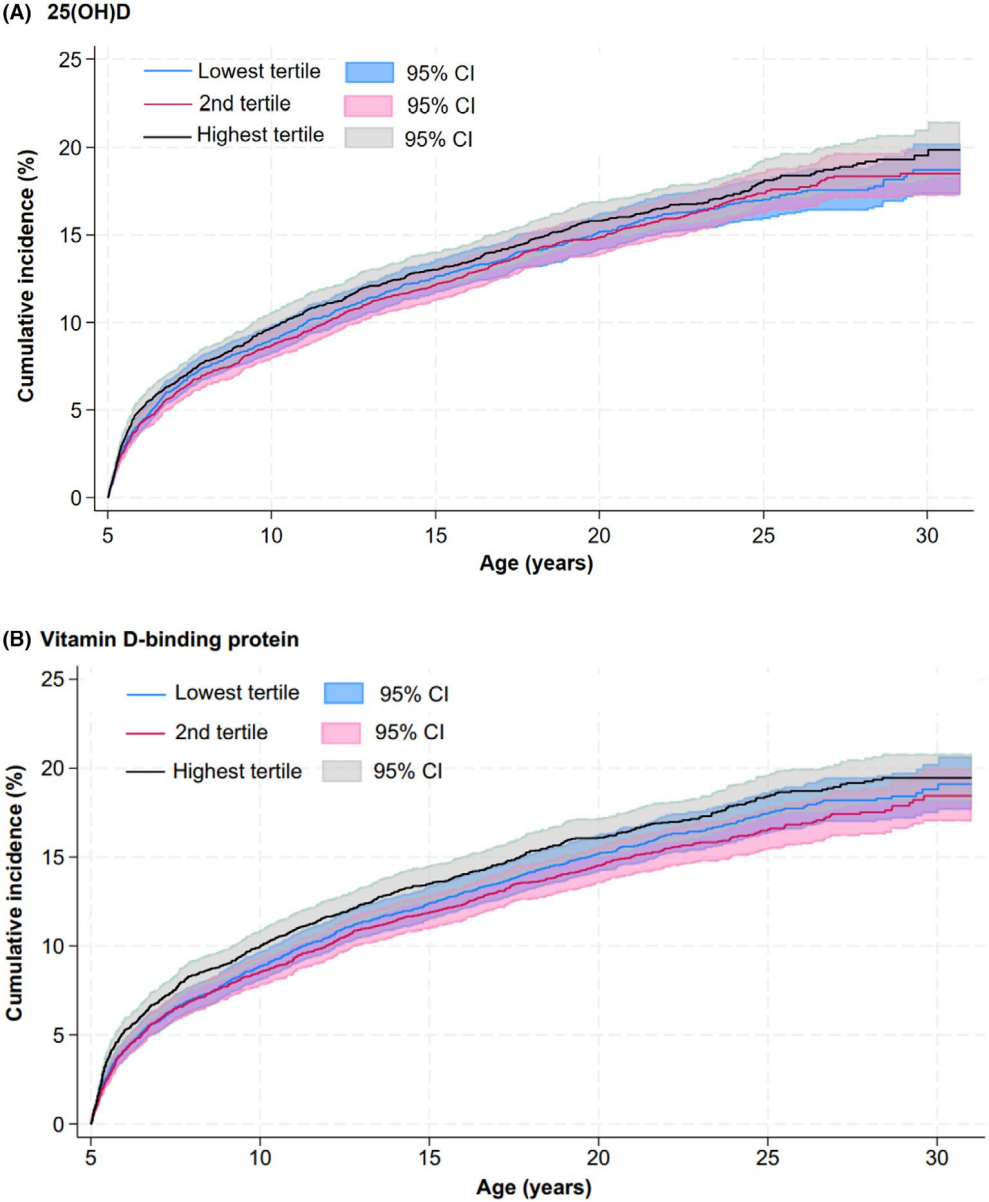
Cumulative incidence of asthma according to tertiles of 25(OH)D and vitamin D‐binding protein.

**FIGURE 3:**
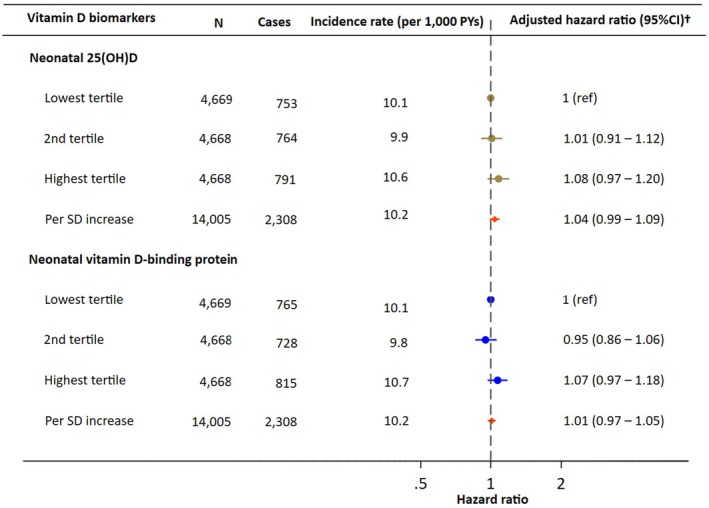
Hazard ratio for asthma by the tertiles and per standard deviation (SD) increase in neonatal 25(OH)D levels and vitamin D‐binding protein levels. ^†^Adjusted for maternal age at delivery, European ancestry, parity, maternal hospital asthma diagnosis before delivery, cohabiting status at delivery, highest education attained, season of birth, gender, and calendar year of birth.

For neonatal 25(OH)D, the cumulative incidence of asthma was similar across tertiles, with overlapping estimates and no clear dose–response relationship: 18.7% (95% CI: 17.3%–20.2%) in the lowest tertile, 18.5% (95% CI: 17.3%–19.8%) in the second tertile, and 19.9% (95% CI: 18.3%–21.5%) in the highest tertile (Figure 2A). These corresponded to adjusted HRs of 1.01 (95% CI: 0.91–1.12) for the second tertile and 1.08 (95% CI: 0.97–1.20) for the highest tertile, compared with the lowest tertile (Figure 3). When 25(OH)D was analyzed as a continuous variable, there was no association with asthma risk, with an HR of 1.04 (95% CI: 0.99–1.09) per SD increase.

For neonatal DBP levels, the cumulative incidence of asthma was also comparable across tertiles, with overlap in the estimates: 19.1% (95% CI: 17.7%–20.7%) in the lowest tertile, 18.5% (95% CI: 17.0%–20.0%) in the second tertile, and 19.5% (95% CI: 18.2%–20.8%) in the highest tertile (Figure 2B). The adjusted HRs were 0.95 (95% CI: 0.86–1.06) for the second tertile and 1.07 (95% CI: 0.97–1.18) for the highest tertile, compared to the lowest tertile (Figure 3). When modeled as a continuous variable, DBP also showed no association with asthma risk, with an HR of 1.01 (95% CI: 0.97–1.05) per SD increase.

### 3.2 Neonatal PGSs for asthma, 25(OH)D, and DBP and asthma risk

**FIGURE 4:**
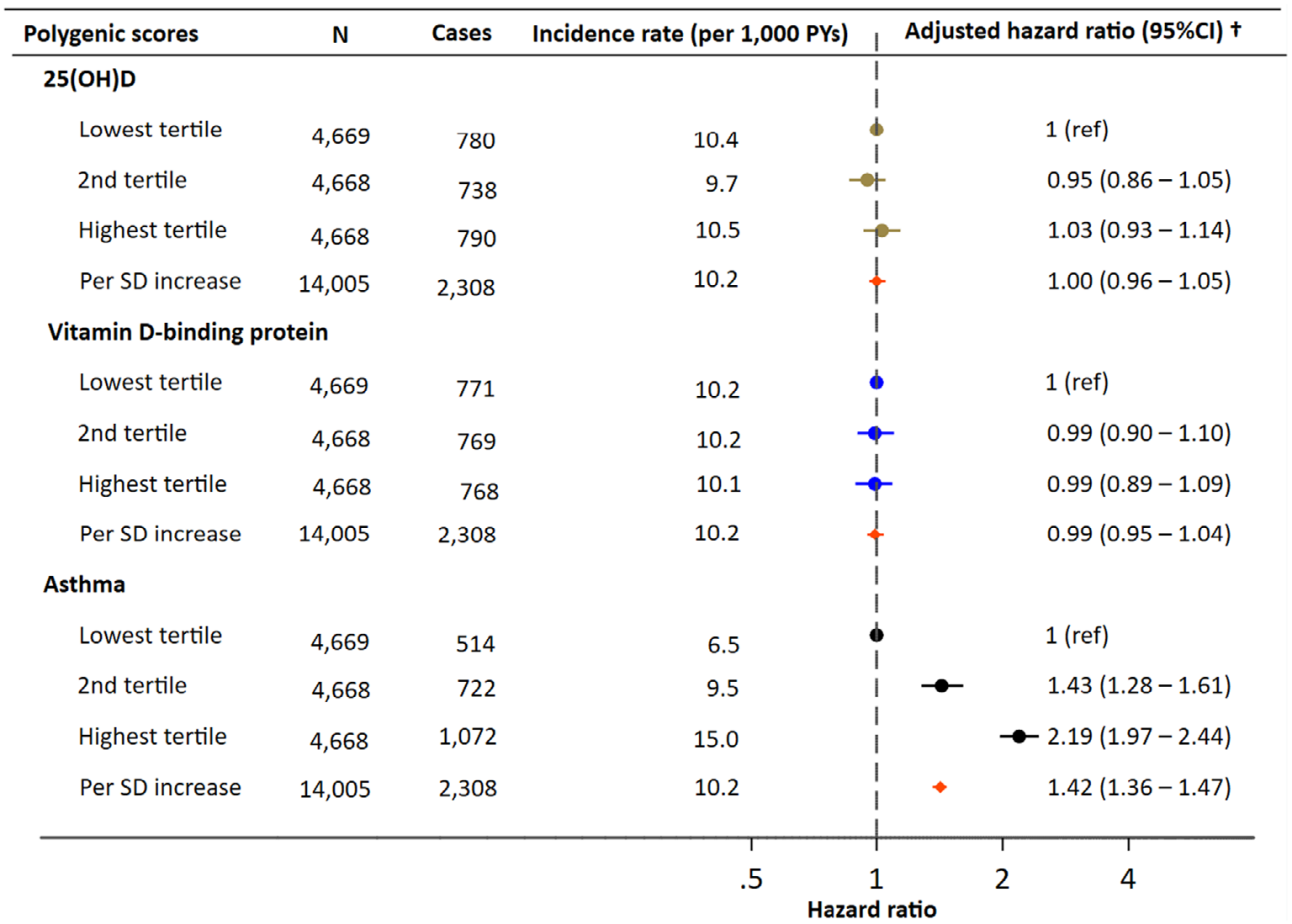
Hazard ratio for asthma by polygenic scores for 25(OH)D, vitamin D‐binding protein, and asthma. ^†^Adjusted for maternal age at delivery, European ancestry, parity, maternal hospital asthma diagnosis before delivery, cohabiting status at delivery, highest education attained, season of birth, gender, calendar year of birth, and the first 10 principal components.

As expected, we observed an association between the asthma PGS and asthma risk, with an HR of 1.42 (95% CI: 1.36–1.47) per SD increase (Figure 4). A clear dose–response relationship was seen across tertiles of asthma PGS. However, no association was found between asthma risk and either genetically‐predicted 25(OH)D or DBP, with HRs of 1.00 (95% CI: 0.96–1.05) and 0.99 (95% CI: 0.95–1.04) per SD increase, respectively. Furthermore, analyses of the additive interaction between neonatal vitamin D levels and asthma PGS on asthma risk showed no evidence of interaction, as reflected by RERI values and their 95% confidence intervals, which included zero (Table S4). The distributions of three PGSs and the cumulative incidence of asthma by each PGS are presented in Figures S4 and S5.

### 3.3 Sensitivity analysis

Sensitivity analyses were conducted separately for individuals of European and non‐European ancestry (see Table S5). Additionally, we performed separate analyses for childhood‐onset asthma (diagnosed by age 20 years, accounting for 90.6% of our cases) and adult‐onset asthma (see Table S6). The results were consistent with the main findings across all subgroups: neither neonatal 25(OH)D levels nor DBP levels were associated with an increased risk of asthma.

### 3.4 Post‐hoc analysis

We examined the relationship between neonatal vitamin D levels and transient wheezing that developed in the first 5 years. The results indicated that neither neonatal 25(OH)D levels nor DBP levels were associated with transient wheezing before age 5 years (see Figure S6).

## 4 DISCUSSION

In this population‐based cohort of 14,005 individuals followed for up to 25 years, we investigated the risk of asthma after age 5 years in relation to two vitamin D biomarkers, 25(OH)D and DBP, as well as their genetic predictors measured in neonatal dried blood spots. Our analysis showed that neither neonatal biomarker was associated with an increased risk of developing asthma after age 5 years. Consistently, PGSs for 25(OH)D and DBP, which predict the concentration of these biomarkers throughout the lifespan, were not associated with asthma risk. Moreover, we observed no additive interaction of 25(OH)D and DBP with asthma PGS, indicating that the absence of an association between neonatal vitamin D status and asthma risk was consistent across levels of asthma genetic susceptibility. Taken together, our findings do not support the hypothesis that neonatal vitamin D status is associated with the development of asthma after age 5 years.

Our findings are consistent with most previous studies, which have reported no association between maternal or cord blood 25(OH)D levels and asthma risk.^4^, ^12^, ^13^, ^14^, ^15^, ^16^, ^17^, ^18^, ^19^, ^20^, ^21^ However, they contradict studies that suggest an inverse association,^7^, ^8^, ^9^ or an increased risk of asthma associated with higher maternal vitamin D levels.^10^, ^11^ These discrepancies may be partly due to differences in how asthma is defined. It is reported that high‐dose vitamin D supplementation during pregnancy was associated with a 25% reduction in asthma/wheeze risk at age 0–3 years compared to placebo,^43^ but not at age 6 years.^44^, ^45^ In our study, we focused on asthma diagnosed after age 5 years, when the diagnosis is generally more specific, acknowledging that a large number of children with an early‐transient asthma/wheeze phenotype that resolves by age 3 years will not be captured as cases.^46^ In contrast, studies reporting an inverse association defined asthma/wheeze at earlier ages, often before age 5 years.^8^, ^9^ However, in our post‐hoc analysis on early‐life transient wheezing, we found no association between neonatal vitamin D levels and transient wheezing in the first 5 years of life. As suggested by a recent Cochrane review, supplementation during pregnancy may reduce the risk of childhood wheeze, but not asthma, whereas supplementation during childhood has no effect on either outcome.^24^ Another plausible explanation is a U‐shaped relationship between maternal 25(OH)D levels and childhood asthma, with a reduced risk observed only in populations with low vitamin D levels.^22^

We only had access to neonatal measures, and critical windows of exposure related to vitamin D and risk of asthma remain unclear. In contrast, PGSs, which represent genetically predicted levels, provide a more stable proxy for assessing genetic liability. In our study, we did not observe an association of PGSs for 25(OH)D and DBP with asthma. However, one limitation is that the PGS for 25(OH)D captures only a small proportion of its phenotypic variance.^47^ In contrast, the PGS for DBP shows high predictive performance (*R* ^2^ >50%),^38^ suggesting that genetically predicted DBP levels may serve as a more precise measure in the analyses. Taken together, our findings, based on both measured vitamin D status and PGS‐based estimates, suggest that neither circulating 25(OH)D levels nor DBP is associated with individual asthma risk. Although vitamin D supplementation during pregnancy remains recommended for bone health, our results do not support an additional benefit for reducing asthma risk after age 5 years.

### 4.1 Strengths and limitations

Our study has several strengths. First, it has a large sample size with a follow‐up of up to 25 years, which provided sufficient statistical power to detect a 13% risk reduction or greater. Second, we directly measured 25(OH)D and DBP in neonatal blood spots. Third, we applied a triangulation approach by including both biomarkers and PGSs to estimate genetically predicted 25(OH)D and DBP across the lifespan. This enhances the generalizability and reliability of the findings. Finally, our analysis was adjusted for a broad range of potential confounders, including socioeconomic status and genetic factors.

Our study also has several limitations. First, we measured 25(OH)D concentrations only at birth, which may not reflect vitamin D status across the life course or early gestation. Vitamin D concentrations after birth fluctuate over time and are influenced by various factors, including diet, supplementation, sun exposure, and lifestyle. Similarly, 25(OH)D PGS explains only a small proportion of its total variance. Second, although we adjusted for various confounders, we lacked detailed data on lifestyle factors, such as smoke exposure and diets, and therefore, residual confounding cannot be ruled out. Third, asthma was identified based on hospital diagnoses and filled asthma medication prescriptions. Individuals with undiagnosed or untreated asthma may have been misclassified as non‐asthma controls, while those receiving asthma medications for other conditions may have been misclassified as asthma cases. This potential misclassification is likely nondifferential and would bias our results toward the null.

## AUTHOR CONTRIBUTIONS

**Xiaoqin Liu:** Conceptualization; methodology; formal analysis; data curation; writing – original draft; visualization; project administration; writing – review and editing; investigation. **Zhihong Zhu:** Conceptualization; methodology; writing – review and editing; investigation. **Bo Chawes:** Conceptualization; methodology; writing – review and editing. **Klaus Bønnelykke:** Conceptualization; methodology; writing – review and editing; investigation. **Henriette Thisted Horsdal:** Writing – review and editing; investigation. **Esben Agerbo:** Writing – review and editing; investigation; data curation. **Sanne Grundvad Boelt:** Writing – review and editing; investigation. **Nis Borbye‐Lorenzen:** Writing – review and editing; investigation. **Lars Melgaard:** Writing – review and editing; investigation. **Carsten Bøcker Pedersen:** Writing – review and editing; investigation. **John J. McGrath:** Conceptualization; methodology; writing – original draft; supervision; resources; project administration; funding acquisition; investigation; writing – review and editing.

## FUNDING INFORMATION

This project was funded by the Lundbeck Foundation through The Lundbeck Foundation Initiative for Integrative Psychiatric Research (R248–2017–2003; R155–2014–1724; R102‐A9118). JJM received funding from the Danish National Research Foundation (Niels Bohr Professor). The funders had no role in the study design and conduct, data collection, management, analysis, interpretation, manuscript preparation, review, approval, or decision to submit the manuscript for publication.

## CONFLICT OF INTEREST STATEMENT

None.

## Supporting information


Appendix S1.

## Data Availability

According to Danish data protection legislation, individual‐level data cannot be made publicly available; only aggregated data may be presented. All data are stored on a secure platform at Statistics Denmark and are accessible only to authorized personnel. The analytical plan has been registered on the Open Science Framework (OSF) and will be made publicly available upon acceptance of the manuscript.
