# Supplementary material for: Neonatal vitamin D status and asthma risk after age 5 years: A Danish population‐based cohort study
Source: Pediatr Allergy Immunol. 2026 Feb 2;37(2):e70299. doi: 10.1111/pai.70299 (PMC12929039; doi:10.1111/pai.70299)
Supplement: Supplementary file 1 — Appendix S1. [file PAI-37-e70299-s001.docx]

**Table S1.** Characteristics of individuals included and excluded from the analysis

| **Maternal characteristics** | **Included individuals** | **Excluded individuals** |
| --- | --- | --- |
| **N** | 14,005 | 5,299 |
| **Maternal age at delivery** |  |  |
| <25 | 2,392 (17.1) | 873 (16.5) |
| 25–29 | 5,220 (37.3) | 2,060 (38.9) |
| 30–34 | 4,523 (32.3) | 1,631 (30.8) |
| ≥35 | 1,870 (13.4) | 735 (13.9) |
| **Primiparous** | 6,172 (44.1) | 2,343 (44.2) |
| **Maternal cohabitant status** |  |  |
| Married or cohabiting | 12,960 (92.5) | 4,844 (91.4) |
| Single, divorced, or widowed | 1,045 (7.5) | 455 (8.6) |
| **Highest education attained** |  |  |
| Mandatory school | 3,368 (24.0) | 1,255 (23.7) |
| High school or vocational school | 6,338 (45.3) | 2,341 (44.2) |
| College or university | 4,019 (28.7) | 1,498 (28.3) |
| Unknown | 280 (2.0) | 205 (3.9) |
| **Maternal asthma hospital diagnosis before delivery** | 225 (1.6) | 84 (1.6) |
| **Season of birth** |  |  |
| Spring | 3,498 (25.0) | 1,426 (26.9) |
| Summer | 3,808 (27.2) | 1,216 (22.9) |
| Autumn | 3,281 (23.4) | 1,518 (28.6) |
| Winter | 3,418 (24.4) | 1,139 (21.5) |
| **Gender of the individuals** |  |  |
| Males | 7,154 (51.1) | 2,742 (51.7) |
| Females | 6,851 (48.9) | 2,557 (48.3) |
| **Calendar year at birth** |  |  |
| 1991–1995 | 4,278 (30.5) | 2,319 (43.8) |
| 1996–2000 | 4,840 (34.6) | 1,621 (30.6) |
| 2001–2005 | 4,887 (34.9) | 1,359 (25.6) |

**Table S2.** Characteristics by the tertiles of neonatal 25(OH)D levels. Figures are numbers (%).

| **Maternal characteristics** | **Neonatal 25(OH)D levels** | | |
| --- | --- | --- | --- |
|  | **Lowest tertile** | **2^nd^ tertile** | **Highest tertile** |
| **N** | 4,669 | 4,668 | 4,668 |
| **Maternal age at delivery** |  |  |  |
| <25 | 950 (20.3) | 781 (16.7) | 661 (14.2) |
| 25–29 | 1,695 (36.3) | 1,777 (38.1) | 1,748 (37.4) |
| 30–34 | 1,446 (31.0) | 1,497 (32.1) | 1,580 (33.8) |
| ≥35 | 578 (12.4) | 613 (13.1) | 679 (14.5) |
| **Primiparous** | 1,864 (39.9) | 2,031 (43.5) | 2,277 (48.8) |
| **Maternal cohabitant status** |  |  |  |
| Married or cohabiting | 4,259 (91.2) | 4,335 (92.9) | 4,366 (93.5) |
| Single, divorced, or widowed | 410 (8.9) | 333 (7.1) | 302 (6.5) |
| **Highest education attained** |  |  |  |
| Mandatory school | 1,363 (29.2) | 1,110 (23.8) | 895 (19.2) |
| High school or vocational school | 2,019 (43.2) | 2,143 (45.9) | 2,176 (46.6) |
| College or university | 1,139 (24.4) | 1,352 (29.0) | 1,528 (32.7) |
| Unknown | 148 (3.2) | 63 (1.3) | 69 (1.5) |
| **Maternal asthma hospital diagnosis before delivery** | 76 (1.6) | 75 (1.6) | 74 (1.6) |
| **European ancestry** |  |  |  |
| Yes | 3,763 (80.6) | 4,122 (88.3) | 4,282 (91.7) |
| No | 906 (19.4) | 546 (11.7) | 386 (8.3) |
| **Season of birth** |  |  |  |
| Spring | 1,209 (25.9) | 1,252 (26.8) | 1,037 (22.2) |
| Summer | 1,253 (26.8) | 1,140 (24.4) | 1,415 (30.3) |
| Autumn | 1,075 (23.0) | 1,073 (23.0) | 1,133 (24.3) |
| Winter | 1,132 (24.2) | 1,203 (25.8) | 1,083 (23.2) |
| **Gender of the individuals** |  |  |  |
| Males | 2,392 (51.2) | 2,356 (50.5) | 2,406 (51.5) |
| Females | 2,277 (48.8) | 2,312 (49.5) | 2,262 (48.5) |
| **Calendar year at birth** |  |  |  |
| 1991–1995 | 1,415 (30.3) | 1,491 (31.9) | 1,372 (29.4) |
| 1996–2000 | 1,551 (33.2) | 1,695 (36.3) | 1,594 (34.1) |
| 2001–2005 | 1,703 (36.5) | 1,482 (31.7) | 1,702 (36.5) |

**Table S3.** Characteristics by the tertiles of neonatal vitamin D binding protein levels. Figures are numbers (%).

| **Maternal characteristics** | **Neonatal vitamin D binding protein levels** | | |
| --- | --- | --- | --- |
|  | **Lowest tertile** | **2^nd^ tertile** | **Highest tertile** |
| **N** | 4,669 | 4,668 | 4,668 |
| **Maternal age at delivery** |  |  |  |
| <25 | 796 (17.0) | 750 (16.1) | 846 (18.1) |
| 25–29 | 1,743 (37.3) | 1,761 (37.7) | 1,716 (36.8) |
| 30–34 | 1,489 (31.9) | 1,526 (32.7) | 1,508 (32.3) |
| ≥35 | 641 (13.7) | 631 (13.5) | 598 (12.8) |
| **Primiparous** | 2,025 (43.4) | 2,076 (44.5) | 2,071 (44.4) |
| **Maternal cohabitant status** |  |  |  |
| Married or cohabiting | 4,256 (91.2) | 4,346 (93.1) | 4,358 (93.4) |
| Single, divorced, or widowed | 413 (8.8) | 322 (6.9) | 310 (6.6) |
| **Highest education attained** |  |  |  |
| Mandatory school | 1,176 (25.2) | 1,091 (23.4) | 1,101 (23.6) |
| High school or vocational school | 2,081 (44.6) | 2,121 (45.4) | 2,136 (45.8) |
| College or university | 1,308 (28.0) | 1,368 (29.3) | 1,343 (28.8) |
| Unknown | 104 (2.2) | 88 (1.9) | 88 (1.9) |
| **Maternal asthma hospital diagnosis before delivery** | 61 (1.3) | 88 (1.9) | 76 (1.6) |
| **European ancestry** |  |  |  |
| Yes | 3,985 (85.4) | 4,051 (86.8) | 4,131 (88.5) |
| No | 684 (14.6) | 617 (13.2) | 537 (11.5) |
| **Season of birth** |  |  |  |
| Spring | 1,126 (24.1) | 1,245 (26.7) | 1,127 (24.1) |
| Summer | 1,259 (27.0) | 1,257 (26.9) | 1,292 (27.7) |
| Autumn | 1,142 (24.5) | 1,007 (21.6) | 1,132 (24.3) |
| Winter | 1,142 (24.5) | 1,159 (24.8) | 1,117 (23.9) |
| **Gender of the individuals** |  |  |  |
| Males | 2,410 (51.6) | 2,404 (51.5) | 2,340 (50.1) |
| Females | 2,259 (48.4) | 2,264 (48.5) | 2,328 (49.9) |
| **Calendar year at birth** |  |  |  |
| 1991–1995 | 1,458 (31.2) | 1,293 (27.7) | 1,527 (32.7) |
| 1996–2000 | 1,700 (36.4) | 1,404 (30.1) | 1,736 (37.2) |
| 2001–2005 | 1,511 (32.4) | 1,971 (42.2) | 1,405 (30.1) |

**Table S4.** Hazard ratios for asthma according to combinations of neonatal 25(OH)D and vitamin D-binding protein (DBP) with asthma PGS

| **Neonatal 25(OH)D or DBP levels** | **Asthma polygenic scores** | **N** | **Asthma cases** | **Incidence rate (per 1000 person-years)** | **Crude HR (95% CI)** | **Adjusted HR (95% CI) *** | **Relative excess risk due to interaction (95% CI)** |
| --- | --- | --- | --- | --- | --- | --- | --- |
| **25(OH)D level** |  |  |  |  |  |  |  |
| Lowest tertile | Lowest tertile | 1,569 | 163 | 6.2 | 1 (ref) | 1 (ref) |  |
| Lowest tertile | 2^nd^ tertile | 1,526 | 237 | 9.6 | 1.53 (1.26–1.87) | 1.51 (1.23–1.85) |  |
| Lowest tertile | Highest tertile | 1,574 | 353 | 14.8 | 2.32 (1.93–2.79) | 2.26 (1.87–2.73) |  |
| 2^nd^ tertile | Lowest tertile | 1,542 | 172 | 6.5 | 1.06 (0.85–1.31) | 1.05 (0.85–1.30) |  |
| 2^nd^ tertile | 2^nd^ tertile | 1,554 | 250 | 9.8 | 1.58 (1.30–1.93) | 1.55 (1.27–1.90) | -0.01 (-0.50–0.48) |
| 2^nd^ tertile | Highest tertile | 1,572 | 342 | 13.7 | 2.20 (1.83–2.65) | 2.15 (1.78–2.59) | -0.16 (-0.79–0.47) |
| Highest tertile | Lowest tertile | 1,558 | 179 | 6.9 | 1.11 (0.90–1.38) | 1.12 (0.90–1.38) |  |
| Highest tertile | 2^nd^ tertile | 1,588 | 235 | 9.1 | 1.46 (1.19–1.78) | 1.47 (1.20–1.80) | -0.16 (-0.65–0.33) |
| Highest tertile | Highest tertile | 1,522 | 377 | 16.5 | 2.59 (2.15–3.11) | 2.56 (2.13–3.09) | 0.18 (-0.50–0.86) |
| **DBP levels** |  |  |  |  |  |  |  |
| Lowest tertile | Lowest tertile | 1,553 | 167 | 6.3 | 1 (ref) | 1 (ref) |  |
| Lowest tertile | 2^nd^ tertile | 1,552 | 251 | 9.9 | 1.55 (1.28–1.89) | 1.53 (1.26–1.86) |  |
| Lowest tertile | Highest tertile | 1,564 | 347 | 14.5 | 2.23 (1.85–2.68) | 2.17 (1.80–2.62) |  |
| 2^nd^ tertile | Lowest tertile | 1,550 | 168 | 6.6 | 1.03 (0.83–1.27) | 1.01 (0.81–1.25) |  |
| 2^nd^ tertile | 2^nd^ tertile | 1,579 | 212 | 8.2 | 1.28 (1.05–1.57) | 1.29 (1.05–1.58) | -0.25 (-0.70–0.20) |
| 2^nd^ tertile | Highest tertile | 1,539 | 348 | 14.9 | 2.28 (1.89–2.73) | 2.23 (1.85–2.68) | 0.05 (-0.57–0.67) |
| Highest tertile | Lowest tertile | 1,566 | 179 | 6.7 | 1.06 (0.86–1.31) | 1.08 (0.87–1.33) |  |
| Highest tertile | 2^nd^ tertile | 1,537 | 259 | 10.3 | 1.62 (1.34–1.97) | 1.61 (1.33–1.96) | 0 (-0.49–0.49) |
| Highest tertile | Highest tertile | 1,565 | 377 | 15.6 | 2.41 (2.01–2.89) | 2.37 (1.98–2.85) | 0.12 (-0.52–0.76) |

Relative excess risk due to interaction is calculated based on the formula: RERIij​=RRij​−RR_i0​−_RR_0j_​+1

* Adjusted for maternal age at delivery, European ancestry, parity, maternal hospital asthma diagnosis before delivery, cohabiting status at delivery, highest education attained, season of birth, gender, calendar year of birth, and the first 10 principal components.

**Table S5.** Hazard ratios of asthma according to neonatal 25(OH)D and vitamin D binding protein (DBP) levels in individuals born of European and non-European ancestry

| **Neonatal 25(OH)D level** | **N** | **Asthma cases** | **Incidence rate (per 1000 person-years)** | **Crude HR (95% CI)** | **Adjusted HR (95% CI) *** |
| --- | --- | --- | --- | --- | --- |
| **European ancestry** |  |  |  |  |  |
| **25(OH)D levels** |  |  |  |  |  |
| Lowest tertile | 3,763 | 606 | 9.9 | 1 (ref) | 1 (ref) |
| 2^nd^ tertile | 4,122 | 672 | 9.8 | 1.01 (0.90–1.12) | 1.01 (0.91–1.13) |
| Highest tertile | 4,282 | 728 | 10.6 | 1.07 (0.96–1.19) | 1.10 (0.99–1.23) |
| Per SD increase | 12,167 | 2,006 | 10.6 | 1.04 (0.99–1.08) | 1.05 (1.00–1.10) |
| **DBP levels** |  |  |  |  |  |
| Lowest tertile | 3,985 | 661 | 10.1 | 1 (ref) | 1 (ref) |
| 2^nd^ tertile | 4,051 | 627 | 9.6 | 0.94 (0.84–1.04) | 0.95 (0.85–1.06) |
| Highest tertile | 4,131 | 718 | 10.6 | 1.05 (0.94–1.17) | 1.05 (0.95–1.17) |
| Per SD increase | 12,167 | 2,006 | 10.6 | 1.00 (0.95–1.04) | 1.00 (0.96–1.04) |
| **Non-European ancestry** |  |  |  |  |  |
| **25(OH)D levels** |  |  |  |  |  |
| Lowest tertile | 906 | 147 | 10.9 | 1 (ref) | 1 (ref) |
| 2^nd^ tertile | 546 | 92 | 10.9 | 1.01 (0.78–1.30) | 0.98 (0.74–1.30) |
| Highest tertile | 386 | 63 | 10.6 | 0.99 (0.74–1.33) | 0.91 (0.66–1.25) |
| Per SD increase | 1,838 | 302 | 10.8 | 1.01 (0.90–1.13) | 0.98 (0.87–1.10) |
| **DBP levels** |  |  |  |  |  |
| Lowest tertile | 684 | 104 | 10.0 | 1 (ref) | 1 (ref) |
| 2^nd^ tertile | 617 | 101 | 10.9 | 1.09 (0.83–1.43) | 1.02 (0.76–1.37) |
| Highest tertile | 537 | 97 | 11.8 | 1.19 (0.90–1.57) | 1.22 (0.91–1.63) |
| Per SD increase | 1,838 | 302 | 10.8 | 1.08 (0.96–1.20) | 1.08 (0.96–1.22) |

* Adjusted for maternal age at delivery, European ancestry, parity, maternal hospital asthma diagnosis before delivery, cohabiting status at delivery, highest education attained, season of birth, gender, and calendar year of birth.

**Table S6.** Hazard ratios of child-onset (<=20 years) and adult-onset asthma according to neonatal 25(OH)D and vitamin D binding protein (DBP) levels

| **Neonatal 25(OH)D level** | **N** | **Asthma cases** | **Incidence rate (per 1000 person-years)** | **Crude HR (95% CI)** | **Adjusted HR (95% CI) *** |
| --- | --- | --- | --- | --- | --- |
| **Child-onset asthma** |  |  |  |  |  |
| **25(OH)D levels** |  |  |  |  |  |
| Lowest tertile | 4,669 | 690 | 11.5 | 1 (ref) | 1 (ref) |
| 2^nd^ tertile | 4,668 | 679 | 11.2 | 0.98 (0.88–1.09) | 0.98 (0.88–1.09) |
| Highest tertile | 4,668 | 721 | 12.1 | 1.05 (0.94–1.16) | 1.06 (0.96–1.19) |
| Per SD increase | 14,005 | 2,090 | 11.6 | 1.03 (0.99–1.08) | 1.04 (0.99–1.09) |
| **DBP levels** |  |  |  |  |  |
| Lowest tertile | 4,669 | 690 | 11.5 | 1 (ref) | 1 (ref) |
| 2^nd^ tertile | 4,668 | 662 | 11.0 | 0.96 (0.86–1.06) | 0.96 (0.86–1.07) |
| Highest tertile | 4,668 | 738 | 12.3 | 1.08 (0.97–1.19) | 1.08 (0.97–1.20) |
| Per SD increase | 14,005 | 2,090 | 11.6 | 1.01 (0.97–1.06) | 1.01 (0.97–1.06) |
| **Adult-onset asthma** |  |  |  |  |  |
| **25(OH)D levels** |  |  |  |  |  |
| Lowest tertile | 2,726 | 63 | 4.2 | 1 (ref) | 1 (ref) |
| 2^nd^ tertile | 2,963 | 85 | 5.2 | 1.24 (0.89–1.71) | 1.27 (0.90–1.79) |
| Highest tertile | 2,728 | 70 | 4.7 | 1.13 (0.80–1.58) | 1.25 (0.87–1.80) |
| Per SD increase | 8,417 | 218 | 4.7 | 1.00 (0.88–1.15) | 1.06 (0.92–1.22) |
| **DBP levels** |  |  |  |  |  |
| Lowest tertile | 2,750 | 75 | 4.8 | 1 (ref) | 1 (ref) |
| 2^nd^ tertile | 2,804 | 66 | 4.6 | 0.97 (0.70–1.35) | 0.90 (0.64–1.27) |
| Highest tertile | 2,863 | 77 | 4.7 | 0.99 (0.72–1.36) | 1.00 (0.72–1.38) |
| Per SD increase | 8,417 | 218 | 4.7 | 0.98 (0.87–1.10) | 0.98 (0.87–1.11) |

* Adjusted for maternal age at delivery, European ancestry, parity, maternal hospital asthma diagnosis before delivery, cohabiting status at delivery, highest education attained, season of birth, gender, and calendar year of birth.

**Figure S1.** Lexis plot outlining the study population and the start and end of follow-up

**
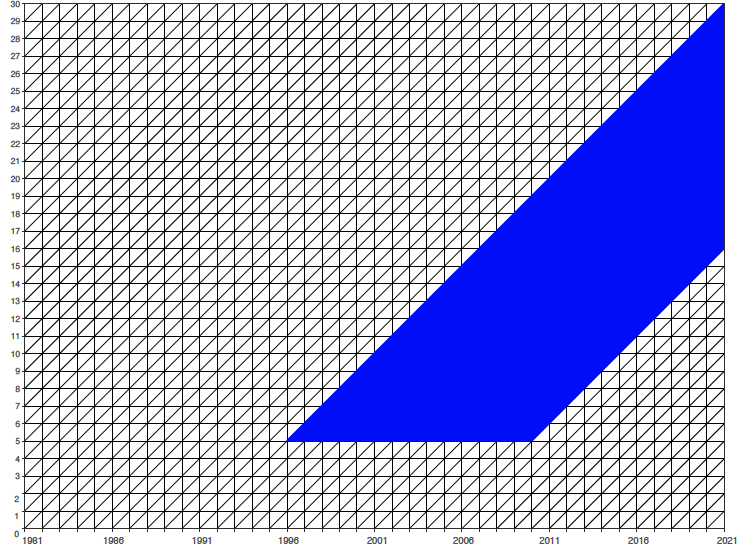
**

**Calendar year of birth**

**Age (years)**

**Figure S2.** Distribution of neonatal 25(OH)D after rank-based inverse normal transformation


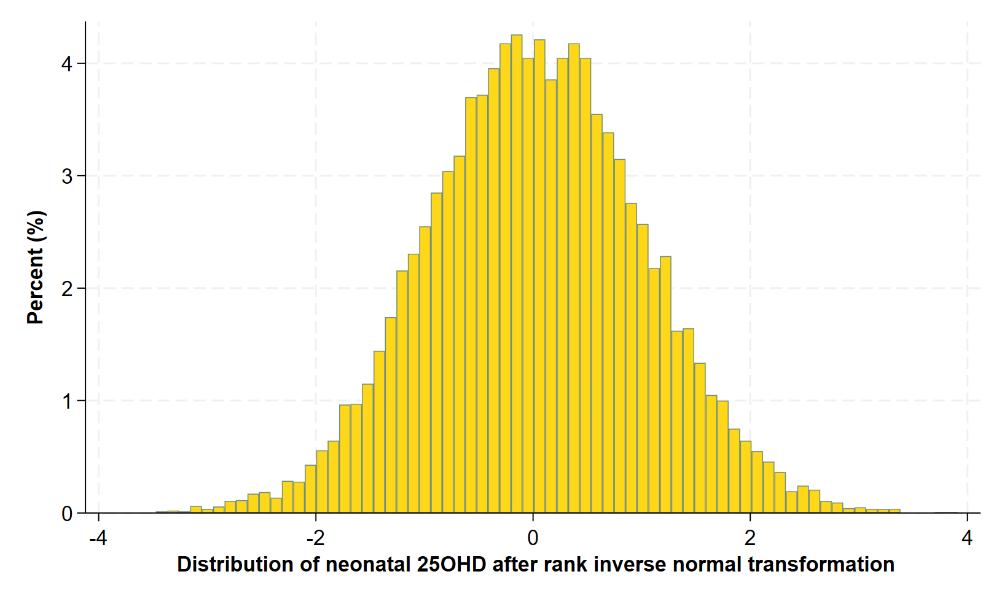


**Figure S3.** Distribution of neonatal DBP after rank-based inverse normal transformation


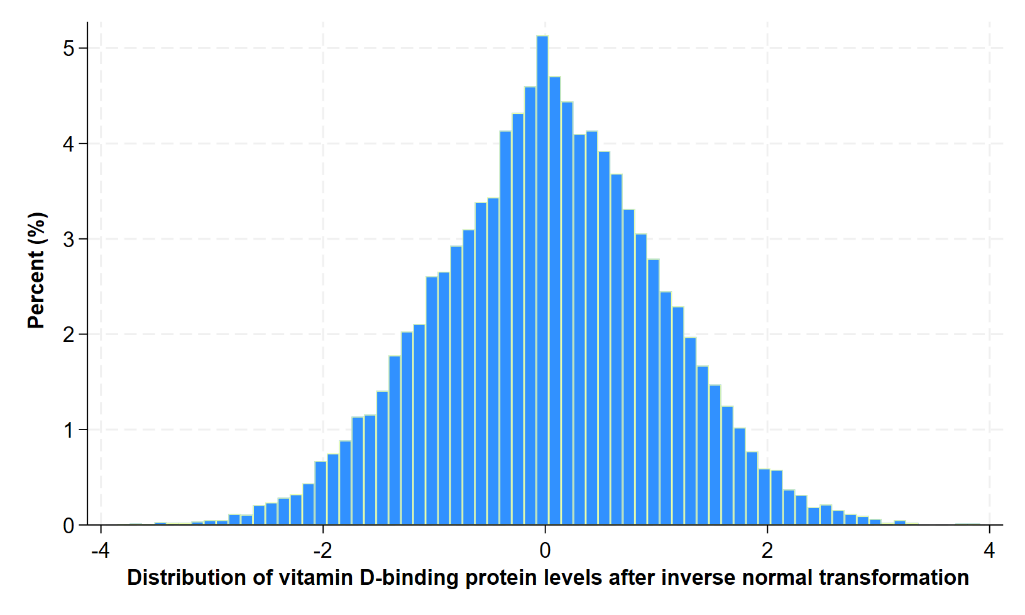


**Figure S4.** Distribution of standardized polygenic scores for 25(OH)D, vitamin D binding protein, and asthma


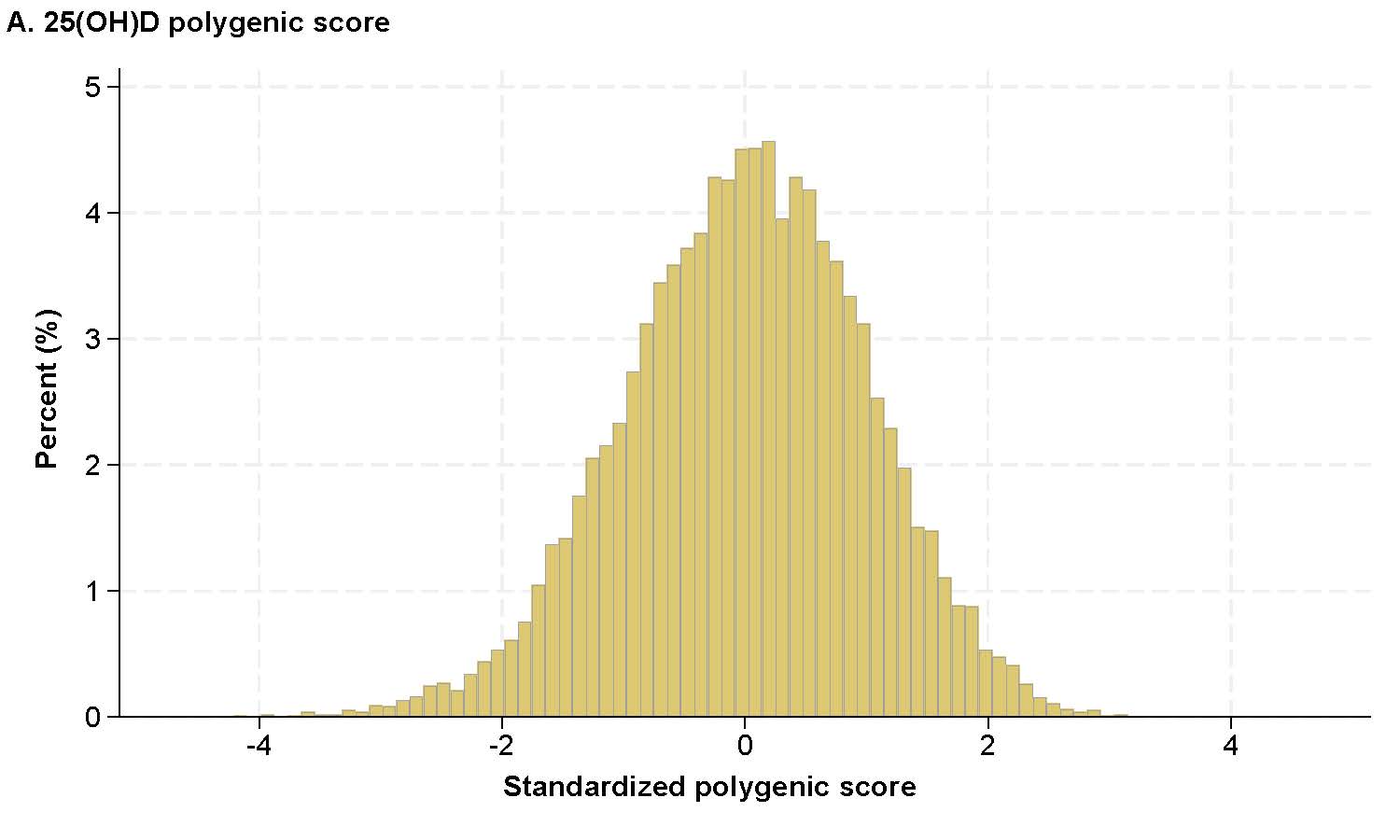


**
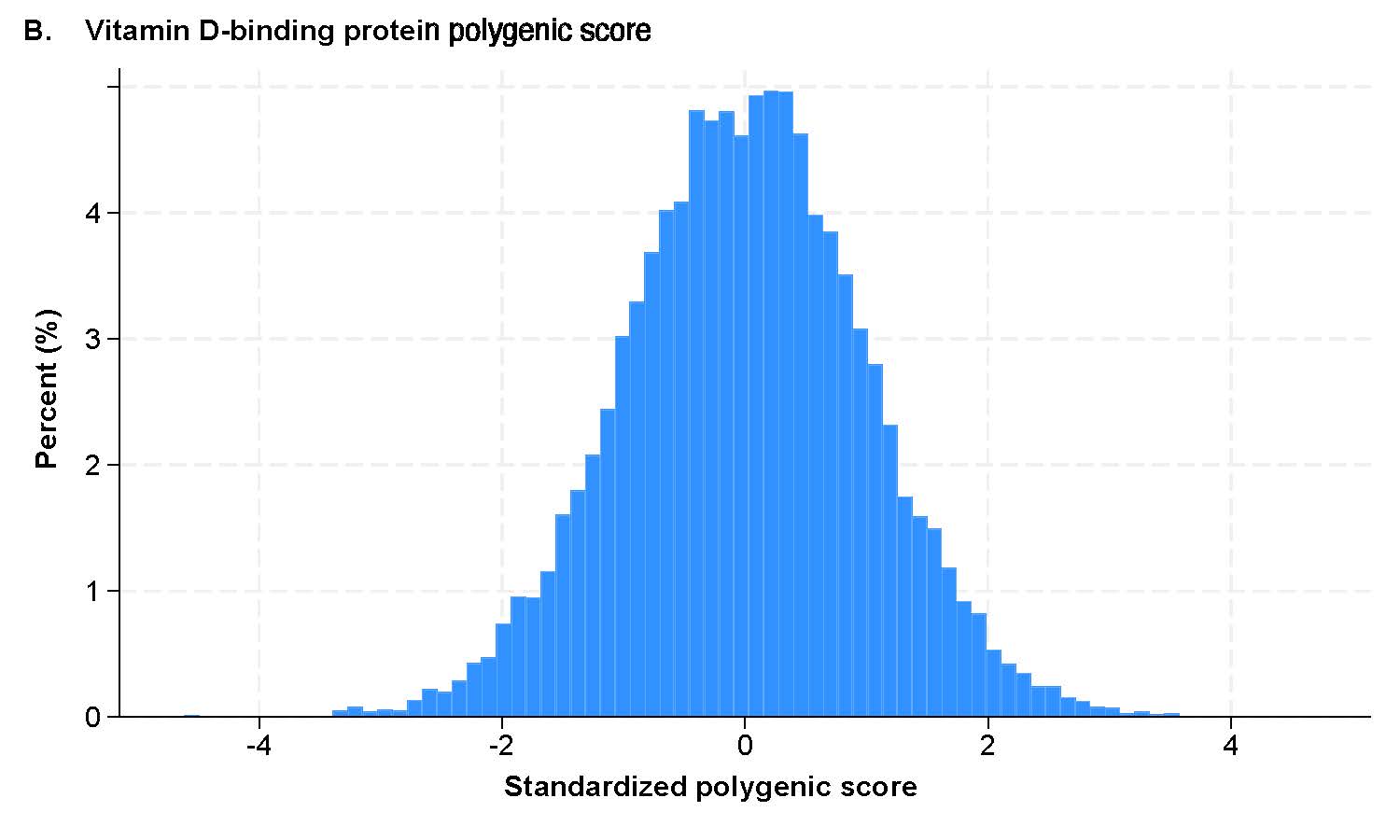
**

**
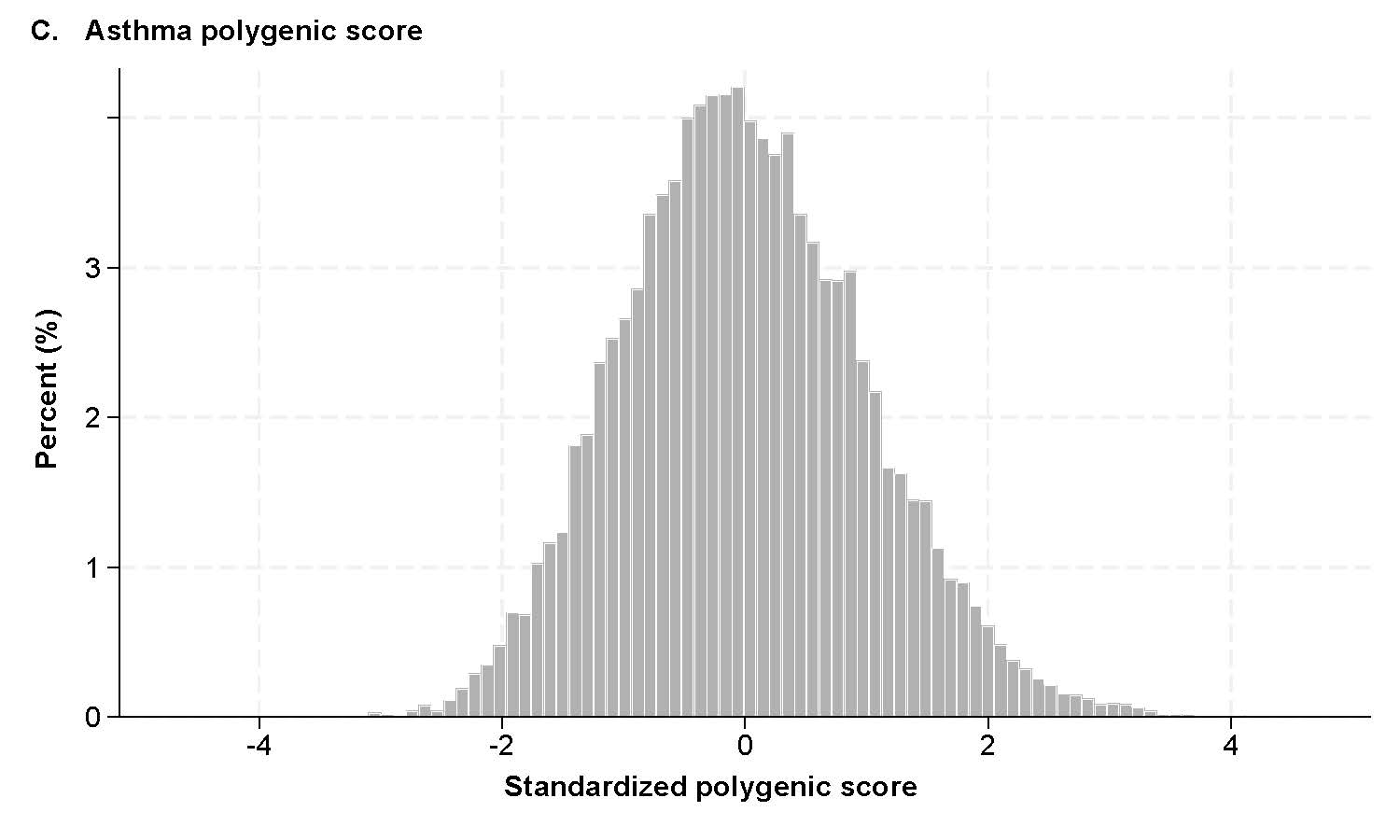
**

**Figure S5.** Cumulative incidence of asthma by tertiles of polygenic scores for 25(OH)D, vitamin D binding protein, and asthma


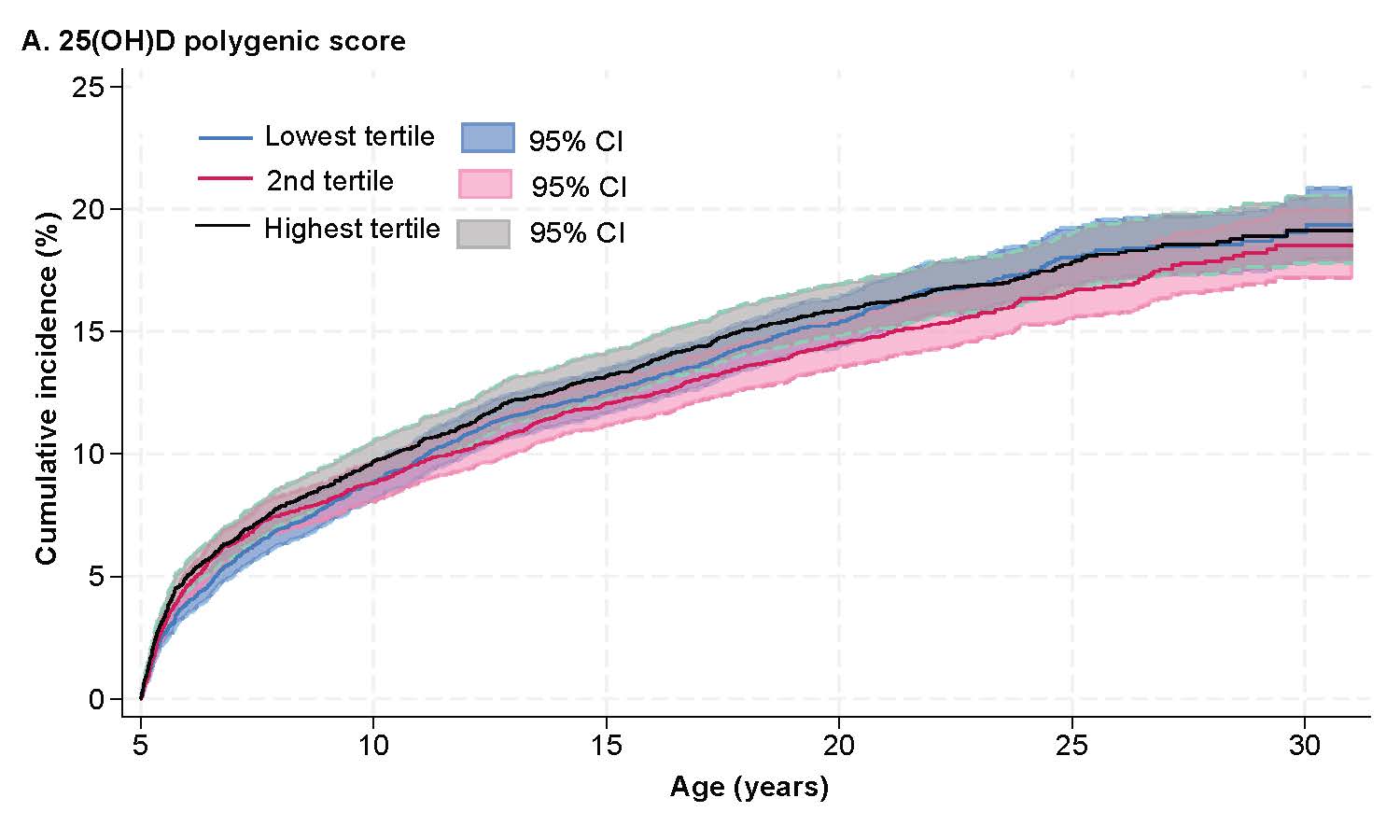


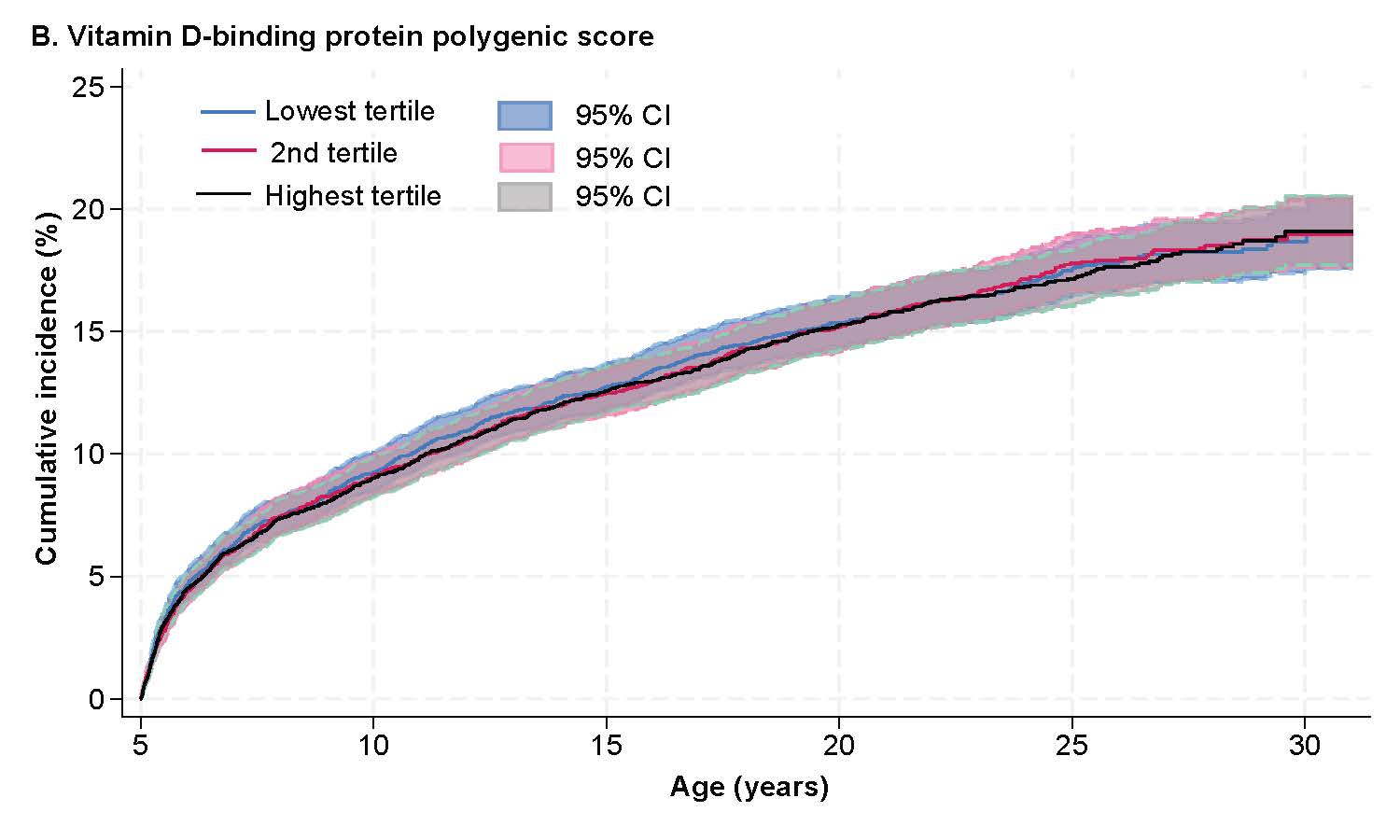

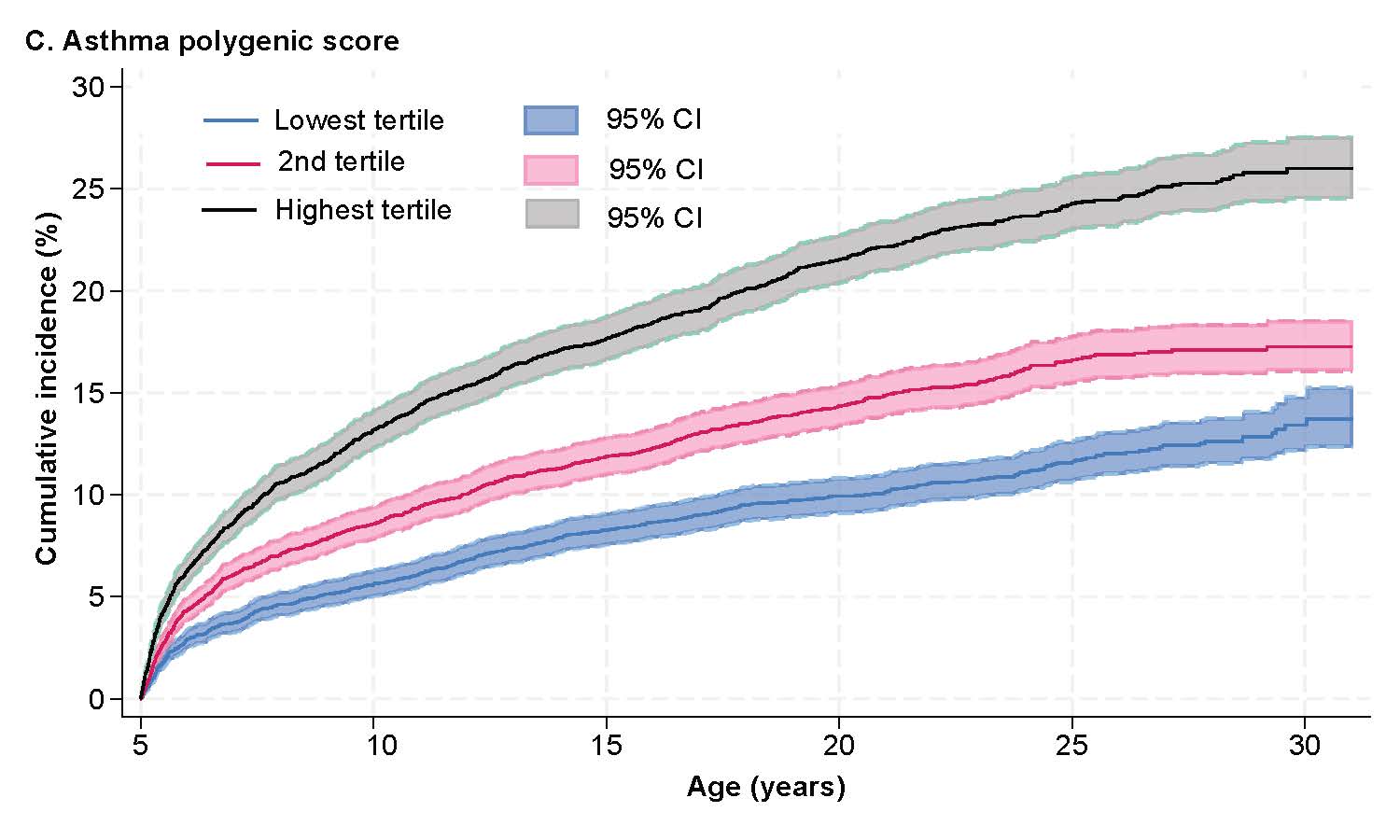


**Figure S6.** Hazard ratio for ***transient wheezing before age 5 years*** by the tertiles of neonatal 25(OH)D levels and vitamin D-binding protein levels

**
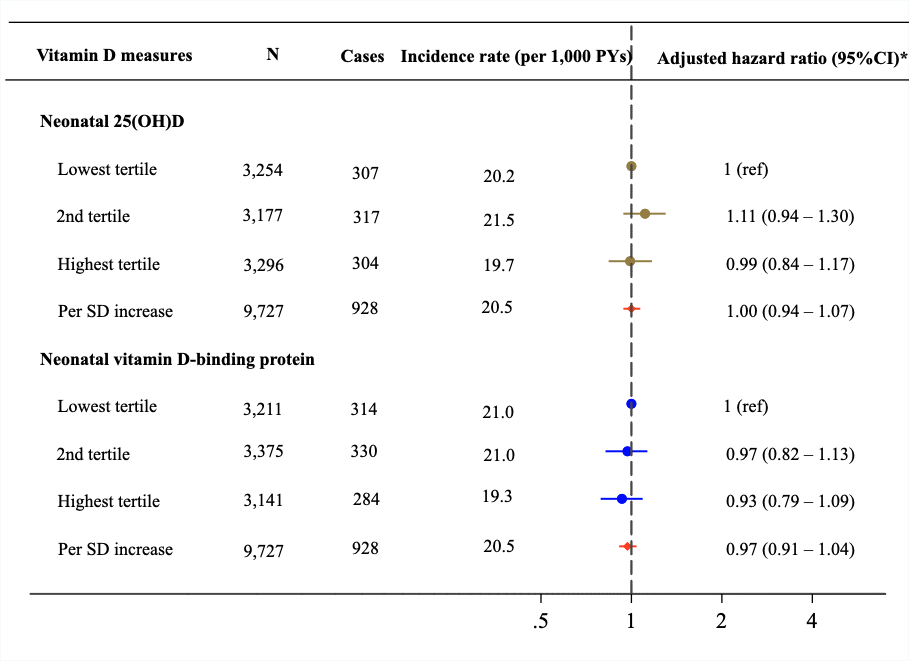
**

Transient wheezing was defined as fulfilling at least one of the criteria A-C before age 5 years, but none of A-C after age 5 years.

* Adjusted for maternal age at delivery, European ancestry, parity, maternal hospital asthma diagnosis before delivery, cohabiting status at delivery, highest education attained, season of birth, and calendar year of birth.
